# Quasi-robust control of biochemical reaction networks via stochastic morphing

**DOI:** 10.1098/rsif.2020.0985

**Published:** 2021-04-14

**Authors:** Tomislav Plesa, Guy-Bart Stan, Thomas E. Ouldridge, Wooli Bae

**Affiliations:** Department of Bioengineering, Imperial College London, Exhibition Road, London SW7 2AZ, UK

**Keywords:** stochastic biochemical reaction networks, timescale separation, control, robustness, synthetic biology, DNA computing

## Abstract

One of the main objectives of synthetic biology is the development of molecular controllers that can manipulate the dynamics of a given biochemical network that is at most partially known. When integrated into smaller compartments, such as living or synthetic cells, controllers have to be calibrated to factor in the intrinsic noise. In this context, biochemical controllers put forward in the literature have focused on manipulating the mean (first moment) and reducing the variance (second moment) of the target molecular species. However, many critical biochemical processes are realized via higher-order moments, particularly the number and configuration of the probability distribution modes (maxima). To bridge the gap, we put forward the *stochastic morpher* controller that can, under suitable timescale separations, morph the probability distribution of the target molecular species into a predefined form. The morphing can be performed at a lower-resolution, allowing one to achieve desired multi-modality/multi-stability, and at a higher-resolution, allowing one to achieve arbitrary probability distributions. Properties of the controller, such as robustness and convergence, are rigorously established, and demonstrated on various examples. Also proposed is a blueprint for an experimental implementation of stochastic morpher.

## 1. Introduction

Synthetic biology is a growing interdisciplinary field of science and engineering whose aims include control of living cells [1–8] and design of synthetic cells with predefined behaviours [9–13]. Key to achieving such goals are nucleic acids (DNA and RNA molecules). In particular, one approach to controlling living cells is by manipulating the dynamics of nucleic acids in the underlying gene-regulatory networks [2–4,6,7]. On the other hand, a large class of biochemical networks can be dynamically realized using DNA/RNA molecules [14,15], which can then be encapsulated inside vesicles, forming pre-programmed synthetic cells [9–13]. At the centre of this nucleic-acid-based synthetic biology (also called DNA/RNA computing) is the highly programmable toehold-mediated strand-displacement mechanism, involving a single-stranded nucleic acid displacing another one from a duplex in accordance with the Watson–Crick base-pairing [14,16–18]. Strand-displacement reactions also play important roles inside living cells [19–21], allowing one to interface synthetic and intracellular DNA/RNA systems [6,7].

**Figure 1.**
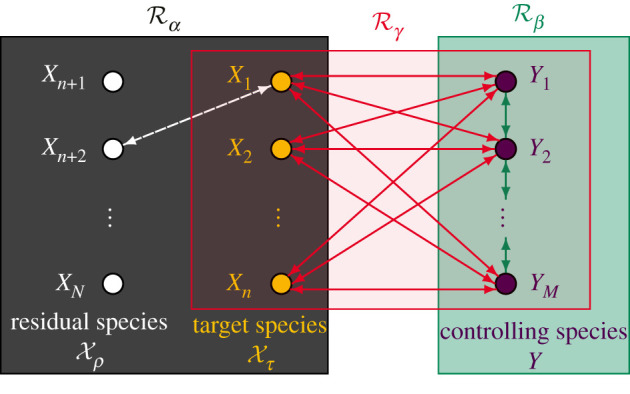
Schematic representation of biochemical control. An *input* network, $R_{\alpha }=R_{\alpha }(X)$, is shown in black. The input species $X=\{X_{1},X_{2},\ldots ,X_{N}\}$ are divided into two mutually exclusive sets: the *target* species $X_{\tau }=\{X_{1},X_{2},\ldots ,X_{n}\}$, that can be explicitly (directly) targeted by the controller and are shown in yellow, and the *residual* species $X_{\rho }=\{X_{n+1},X_{n+2},\ldots ,X_{N}\}$, that can be only implicitly (indirectly) affected by the controller and are shown in white. The coupling between the input species is unknown (respectively, is only partially known) for a *black-box* (respectively, *grey-box*) input network. A known coupling between *X*_1_ and *X*_*n*+2_ is depicted as a white dashed double-arrow. A *controller* is shown, consisting of the sub-network $R_{\beta }=R_{\beta }(Y)$, called the *core*, and $R_{\gamma }=R_{\gamma }(X_{\tau },Y)$, called the *interface*, displayed in green and red, respectively. The controller core $R_{\beta }$ specifies how the *controlling* species $Y=\{Y_{1},Y_{2},\ldots ,Y_{M}\}$, introduced by the controller and shown in purple, interact among themselves, and is depicted as the green double-arrows. On the other hand, the controller interface $R_{\gamma }$ specifies how the controlling species are coupled with the target species, which is displayed as the red double-arrows. Embedding the controller $R_{\beta ,\gamma }=R_{\beta }\cup R_{\gamma }$ into the input network $R_{\alpha }$ gives rise to the *output* network $R_{\alpha }\cup R_{\beta }\cup R_{\gamma }$.

Manipulating the dynamics of cells *in vivo*, and designing their synthetic counterparts *in vitro*, are complementary goals of synthetic biology, both involving overcoming nonlinear, non-modular and stochastic nature of biochemical networks [2]. In particular, when biochemical systems are integrated into smaller-volume compartments, such as living or synthetic cells, the lower copy-numbers of some of the underlying species give rise to intrinsic noise [9–11,22–26]. The induced stochasticity requires theoretical and experimental methods (see section S1 in the electronic supplementary material and §5, respectively) which are more involved than their larger-volume (deterministic) counterparts [14,15,27]. In this context, a central problem is the development of a *controller* network that, when embedded into a given *input* network, ensures that the resulting *output* network executes a predefined stochastic dynamics in a stable and accurate manner, see figure 1. Importantly, the structure and dynamics of the input network are either unknown (*black-box*) or only partially known (*grey-box*). Stochastic control can be sought over probability distributions of the desired biochemical species (*weak control*), or at the level of the underlying sample paths (*strong control*). See also section S2 in the electronic supplementary material, where we rigorously formulate concepts for stochastic biochemical control.

Molecular controllers must satisfy a set of constraints arising from biochemistry in order to be experimentally realizable. At the structural level, controllers should consist of up to second-order (bi-molecular) reactions [28]; at the kinetic level, the underlying reaction rates should be tunable over a sufficiently large range. At the dynamical level, assuming stability, a controller is said to be *robust* if the dynamics of desired species in the output network does not explicitly depend on the initial conditions, nor on the rate coefficients from the input network; if these two conditions are satisfied only up to an arbitrarily small error under a suitable timescale separation, then the controller is said to be *quasi-robust*, see section S2 in the electronic supplementary material for more details. (Quasi-) robustness ensures that the output network accurately traces the predefined dynamics even when the initial conditions are unknown or experimentally difficult to manipulate. For example, the division of a living cell induces extrinsic noise into the initial conditions of the daughter cells [22]; analogously, designing synthetic cells can involve dividing biochemical systems from test-tubes into a larger number of cell-like vesicles with a limited accuracy [9–11]. (Quasi-)robustness also ensures that the predefined dynamics can be achieved by fine-tuning the rate coefficients in the controller without having to know the unknown and uncontrollable rate coefficients from a black-box input network. Dynamics achieved under (quasi-) robust controllers are said to display (*quasi*-)*robust adaptation*, and plays important roles in biology, e.g. in cell signalling, glycolysis and chemokinesis [29–34]; the same is true for timescale separations (slow-fast dynamics), which underpin biochemical multi-stability, oscillations and bifurcations [22–25,27,35–38].

(Quasi-)robust biochemical controllers developed in the literature have been predominantly focused on manipulating the stationary mean (first-moment) of the target species [39–42] and reducing their stationary variance (second-moment) [43,44]. This approach is a step forward from controlling the deterministic dynamics to which the underlying stochastic dynamics converges in the thermodynamic limit [45]. However, many important biochemical phenomena, such as cellular differentiation and memory, quorum sensing, bacterial chemokinesis and antibiotic resistance, are realized via higher-order moments of the underlying probability distributions [24,25,35,37,46–48]. For example, as a consequence of containing a gene that can stochastically and transiently (reversibly) switch between two different states, some cells can produce a key regulatory protein whose abundance follows a probability distribution with two modes (maxima), with each of the modes giving rise to a distinct transient cell phenotype. The genetic bi-modality thus gives rise to a bi-phenotypic population of cells that can have a higher chance of surviving a changing environment [46]; for example, this phenomenon allows some bacteria to persistently survive antibiotic treatments [48]. In this context, particularly important is the number and configuration of the modes present in the probability distributions of the molecular species, and the timing and pattern of stochastic switching in the underlying sample paths. Such dynamically exotic and biochemically important phenomena cannot be ensured using controllers that target only the mean and variance.

To bridge the gap, we put forward a quasi-robust controller called *stochastic morpher*, presented algorithmically in section S3 in the electronic supplementary material. Stochastic morpher consists of a suitable faster interfacing sub-network de-signed to override the underlying black-box input network and, together with a suitable slower core sub-network, gradually transform (morph) the probability distribution of the desired species into a predened form. This control architecture is based on the phenomenon called noise-induced mixing [25] that some gene-regulatory networks utilize *in vivo* [37]. Control may be achieved at two different levels: the lower-resolution stochastic morpher incorporates simple production and degradation of the target species as its faster sub-network that, in combination with noise-induced mixing, allows one to achieve multi-modal probability distributions with desired number and configuration of the modes (weak control), and with controlled average timing and mode-switching pattern in the underlying multi-stable sample paths (strong control). The higher-resolution stochastic morpher involves a more complicated faster interfacing network, and can achieve arbitrary probability distributions.

The rest of the paper is organized as follows. In §2, we apply stochastic morpher on the one-species test network (2.1). In §3, we focus on the lower-resolution control in greater detail, by explicitly controlling two target species from the three-species test network (3.1). In §4, we apply stochastic morpher on the gene-expression network (4.1), and demonstrate how implicit control can be achieved. In particular, we explicitly influence the mRNA (target species) in a suitable way, ensuring that the translated protein (residual species) is implicitly controlled. In §5, we put forward a blueprint for an experimental realization of stochastic morpher using DNA strand-displacement nanotechnology in order to achieve a bi-phenotypic synthetic cell. Finally, we conclude by presenting a summary and discussion in §6. The notation and background theory utilized in the paper are introduced as needed and are summarized in sections S1–S2 in the electronic supplementary material. General properties of stochastic morpher, outlined via specific examples in §2–4, are rigorously established in Sections S4–S6 in the electronic supplementary material.

## 2. Production–degradation input network

Consider the one-species uni-molecular input network $R_{\alpha }^{1}=R_{\alpha }^{1}(X)$, given by

2.1 $$R_{\alpha }^{1}\;:\;\text{Ø}\overset{\alpha_{1}}{\underset{\alpha_{2}}{⇌}}X,$$

where we adopt the convention of denoting two irreversible reactions (e.g. Ø→X and X→Ø) jointly as a single reversible reaction (e.g. Ø⇌X). In this paper, biochemical species, and their copy-numbers as a function of time *t*, are represented with upper-case letters (such as *X*, and *X*(*t*), respectively), while the copy-number values are denoted by the corresponding lower-case letters (such as *x*). Symbol Ø denotes biochemical species that are not explicitly taken into an account. Furthermore, we assume reaction networks are under mass-action kinetics [49], with the positive dimensionless rate coefficients displayed above/below the reaction arrows.

**Figure 2.**
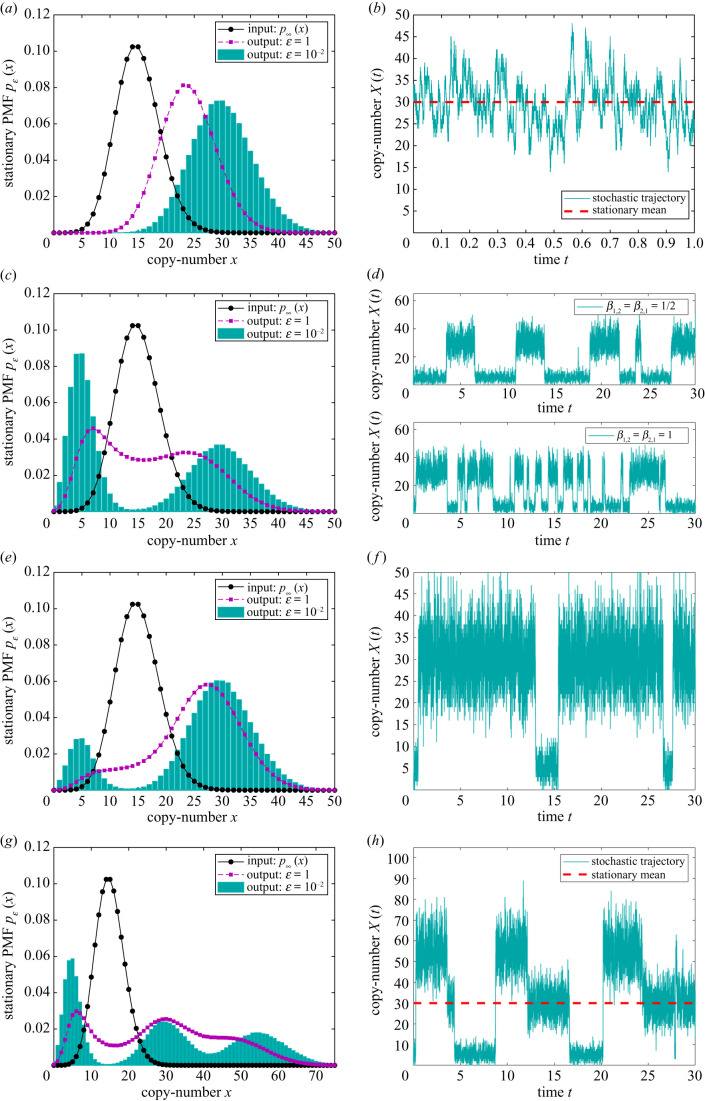
Application of the lower-resolution stochastic morpher on the input network (2.1) with (*α*_1_, *α*_2_) = (1, 1/15). The stationary PMF of the input network is displayed as the interpolated black dots. (*a*) Numerically obtained long-time *x*-marginal PMF of the output networks (2.1) ∪ (2.2) with *β*_1,1_ = 1, (*γ*_0_, *γ*_1_) = (1, 30), and different values of *ɛ*. (*b*) Displays a representative sample path corresponding to the cyan histogram from (*a*), obtained by applying the Gillespie algorithm [50], together with the stationary mean (first moment), shown as a red dashed line. Analogous plots are shown for the output networks (2.1) ∪ (2.4) with (*γ*_0_, *γ*_1_, *γ*_2_) = (1, 5, 30) and (*β*_1,1_, *β*_1,2_, *β*_2,1_) = (1, 1/2, 1/2) (as well as (*β*_1,1_, *β*_1,2_, *β*_2,1_) = (1, 1, 1)) in (*c*,*d*), while with (*β*_1,1_, *β*_1,2_, *β*_2,1_) = (1, 5/6, 1/6) in (*e*,*f*). Finally, (*g*,*h*) show the plots for the output networks (2.1) ∪ (2.6) with (*γ*_0_, *γ*_1_, *γ*_2_, *γ*_3_) = (1, 5, 55, 30) and (*β*_1,1_, *β*_1,2_, *β*_2,3_, *β*_3,1_) = (1, 1/3, 1/3, 1/3).

**Figure 3.**
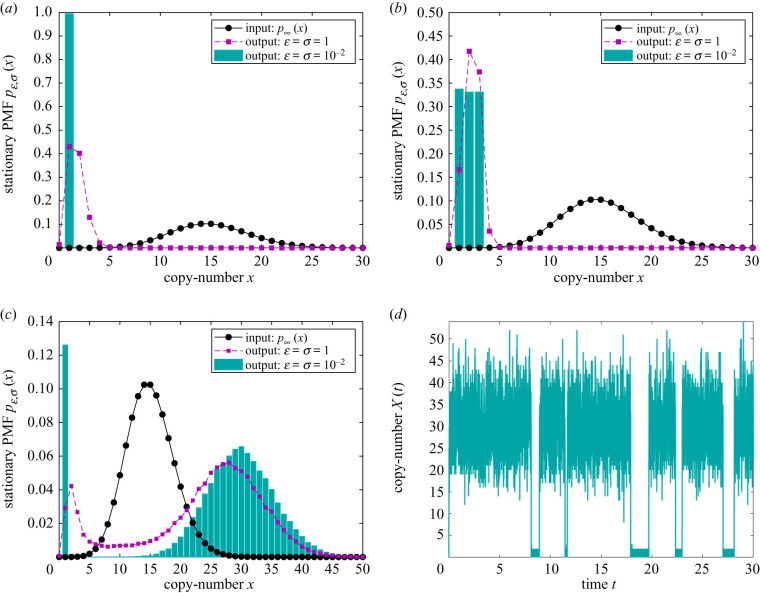
Application of the higher-resolution stochastic morpher on the input network (2.1) with (*α*_1_, *α*_2_) = (1, 1/15). The stationary PMF of the input network is displayed as the interpolated black dots. (*a*) The long-time *x*-marginal PMF of the effective output network (2.1) ∪ (2.8) with *β*_1,1_ = 1, and different values of (*ɛ*, *σ*), which approximates (2.1) ∪ (2.7) as *μ* → 0. (*b*) Displays an analogous plot for the output network (2.1) ∪ (S56) with (*β*_1,1_, *β*_1,2_, *β*_2,3_, *β*_3,1_) = (1, 1/3, 1/3, 1/3). (*c*) The long-time *x*-marginal PMF of the output network (2.1) ∪ (S57) with (*β*_1,1_, *β*_1,2_, *β*_2,1_) = (1, 1/8, 7/8), $\left(\gamma_{1}^{P},\;{\tilde{\gamma }}_{1}^{P})=(30,1\right)$, $\left(\gamma_{0,1}^{\delta },\;\gamma_{0,2}^{\delta },\;\gamma_{2}^{\delta })={\left(\mu^{2}\varepsilon \sigma \right)}^{-1/3}(\mu^{1/3},\;\mu^{-1/6},\mu^{-1/6}\right)$, *μ* = 10^−10^, with (*d*) displaying a corresponding sample path.

In what follows, we fix the (dimensionless) rate coefficients of the input network $R_{\alpha }^{1}(X)$ to ***α*** = (*α*_1_, *α*_2_) = (1, 1/15). The stationary probability-mass function (PMF) of (2.1), describing the long-time dynamics of the input network and denoted by *p*_∞_(*x*), is given by the Poisson distribution with mean (centred at) *α*_1_/*α*_2_ [26], denoted by $p_{\infty }(x)=P(x;\;\alpha_{1}/\alpha_{2})$. For the particular choice of the rate coefficients, the Poisson PMF is centred at *x* = *α*_1_/*α*_2_ = 15 and is shown as the black dots in figures 2 and 3, which are interpolated with solid black lines for visual clarity. In the rest of this section, we embed different forms of stochastic morpher into the input network (2.1) in order to desirably influence the dynamics of the species *X* and showcase the capabilities of the controller. Network (2.1) can be interpreted as a simplified model of genetic transcription, with *X* representing an mRNA species being transcribed and degraded, see §4 for a more-detailed model.

### 2.1. Lower-resolution control

#### 2.1.1. Uni-modality

Consider the stochastic morpher $R_{\beta }\cup R_{\gamma }^{P}=R_{\beta }(Y_{1})\;\cup R_{\gamma }^{P}(X;\;Y_{1})$, given by

2.2 $$\begin{array}{rl} & R_{\beta }\;:\;2Y_{1}\overset{\beta_{1,1}}{⟶}Y_{1},\\ & R_{\gamma }^{P}\;:\;R_{\gamma_{0}}^{\varepsilon }\;:\;X\overset{\gamma_{0}/\varepsilon }{⟶}\text{Ø},\\ & R_{\gamma_{1}}^{\varepsilon }\;:\;Y_{1}\overset{\gamma_{1}/\varepsilon }{⟶}Y_{1}+X, \end{array}$$

where *X* is the target species and *Y*_1_ is the controlling species; see figure 1 for a general set-up. Controller (2.2) consists of two sub-networks: the core network $R_{\beta }\;(Y_{1})$, describing a bi-molecular degradation of *Y*_1_, and the interfacing network $R_{\gamma }^{P}(X;\;Y_{1})=R_{\gamma_{0}}^{\varepsilon }(X;\;\text{Ø})\cup R_{\gamma_{1}}^{\varepsilon }(X;\;Y_{1})$, describing a degradation of *X*, and a production of *X* catalysed by *Y*_1_. To emphasize the catalytic role of *Y*_1_ in $R_{\gamma_{1}}^{\varepsilon }$, we write $R_{\gamma_{1}}^{\varepsilon }=R_{\gamma_{1}}^{\varepsilon }(X;\;Y_{1})$; since $R_{\gamma_{0}}^{\varepsilon }$ is not catalysed by *Y*_1_, we write $R_{\gamma_{0}}^{\varepsilon }=R_{\gamma_{0}}^{\varepsilon }(X;\;\text{Ø})$. Let us note that reaction $R_{\gamma_{0}}^{\varepsilon }$ is implicitly assumed to be of the form *Y*_0_ + *X* → *Y*_0_, where the abundance of an additional controlling species *Y*_0_ is absorbed into an effective rate coefficient *γ*_0_/*ɛ*; see also remarks at the end of this section. The super-script P from $R_{\gamma }^{P}$ stands for the Poisson distribution, as motivated shortly.

In what follows, we analyse the output network $R_{\alpha }^{1}\cup R_{\beta }\cup R_{\gamma }^{P}$, obtained by embedding the stochastic morpher (2.2) into the input network (2.1), which we denote by (2.1) ∪ (2.2). Assuming the copy-number of *Y*_1_ is initially non-zero, the purpose of network $R_{\beta }(Y_{1})$ is to fire until the unit copy-number *y*_1_ = 1 is reached; the stationary marginal PMF of the target species *X* from (2.1)–(2.2) is then given by $p_{\varepsilon }(x)=P(x;\;(\gamma_{1}+\varepsilon \alpha_{1})/(\gamma_{0}+\varepsilon \alpha_{2})),$ implying that

2.3 $$p_{\varepsilon }(x)=\left\{ \begin{array}{cc} P\left(x;\;\frac{\alpha_{1}}{\alpha_{2}}\right), & \mathrm{as}\;\varepsilon \to \infty ,\\ P\left(x;\;\frac{\gamma_{1}}{\gamma_{0}}\right), & \mathrm{as}\;\varepsilon \to 0. \end{array}\right.$$

Therefore, as the interfacing network $R_{\gamma }^{P}$ fires faster, the input network $R_{\alpha }^{1}$ is over-ridden, and the stationary *x*-marginal PMF of the output network $R_{\alpha }^{1}\cup R_{\beta }\cup R_{\gamma }^{P}$ is gradually transformed (morphed) from the Poisson PMF centred at *x* = *α*_1_/*α*_2_ to the Poisson PMF centred at *x* = *γ*_1_/*γ*_0_. Note that this uni-modal morphing controls the first-moment (mean) of the output network. In figure 2*a*, we display the long-time *x*-marginal PMFs for different values of *ɛ*, with the coefficients from $R_{\beta }(Y_{1})$ and $R_{\gamma }^{P}(X;\;Y_{1})$ fixed to *β*_1,1_ = 1 and ***γ*** = (*γ*_0_, *γ*_1_) = (1, 30), respectively. In particular, the long-time PMF is a Poisson distribution centred at *x* = 24 when *ɛ* = 10, shown as the purple squares which, in accordance with (2.3), converges close to the Poisson distribution centred at *x* = *γ*_1_/*γ*_0_ = 30 when *ɛ* = 10^−2^, shown as the cyan histogram in figure 2*a*. A representative sample path corresponding to the histogram is displayed in figure 2*b*, together with the mean which is shown as a red dashed line. The sample path is displayed over a shorter time interval, allowing for the timescale of the underlying fluctuations around the mean to be more discernible.

#### 2.1.2. Bi-modality

Consider now the stochastic morpher $R_{\beta }(Y_{1},Y_{2})\cup R_{\gamma }^{P}(X;Y_{1},Y_{2})$, given by

2.4 $$\begin{array}{rl} & R_{\beta }\;:\;2Y_{1}\overset{\beta_{1,1}}{⟶}Y_{1}\overset{\beta_{1,2}}{\underset{\beta_{2,1}}{⇌}}Y_{2},\\ & R_{\gamma }^{P}\;:\;R_{\gamma_{0}}^{\varepsilon }\;:\;X\overset{\gamma_{0}/\varepsilon }{⟶}\text{Ø},\\ & R_{\gamma_{1}}^{\varepsilon }\;:\;Y_{1}\overset{\gamma_{1}/\varepsilon }{⟶}Y_{1}+X,\\ & R_{\gamma_{2}}^{\varepsilon }\;:\;Y_{2}\overset{\gamma_{2}/\varepsilon }{⟶}Y_{2}+X. \end{array}$$

Sub-network $R_{\beta }(Y_{1},Y_{2})$ describes first-order conversion between the two controlling species *Y*_1_ and *Y*_2_, with the reaction 2*Y*_1_ → *Y*_1_ ensuring that the species *Y*_1_ and *Y*_2_ satisfy the conservation law (*y*_1_ + *y*_2_) = 1 in the long run. On the other hand, sub-network $R_{\gamma }^{P}(X;\;Y_{1},Y_{2})$ involves two production reactions for the species *X*, one catalysed by *Y*_1_ and the other by *Y*_2_. Ignoring the reaction 2*Y*_1_ → *Y*_1_, note that (2.4) can be interpreted as describing a gene switching between two different states *Y*_1_ and *Y*_2_, and producing an mRNA species *X* at different rates [25,37].

When (*y*_1_, *y*_2_) = (1, 0), reaction $R_{\gamma_{2}}^{\varepsilon }$ from (2.4) cannot fire, and the remaining faster reactions generate the Poisson PMF centred at *x* = *γ*_1_/*γ*_0_; analogously, when (*y*_1_, *y*_2_) = (0, 1), the Poisson PMF centred at *x* = *γ*_2_/*γ*_0_ is induced. As the controlling species *Y*_1_ and *Y*_2_ convert between themselves, they mix the two Poisson PMFs and over-ride the input network (2.1); the resulting long-time *x*-marginal PMF of the output network (2.1) ∪ (2.4) is close to

2.5 $$\begin{array}{rl} & q(x)={\left(1+\frac{\beta_{1,2}}{\beta_{2,1}}\right)}^{-1}P\left(x;\;\frac{\gamma_{1}}{\gamma_{0}}\right)+{\left(1+\frac{\beta_{2,1}}{\beta_{1,2}}\right)}^{-1}P\left(x;\;\frac{\gamma_{2}}{\gamma_{0}}\right),\\ & \;\mathrm{as}\;\varepsilon \to 0, \end{array}$$

see theorem S5.1 in section S5 in the electronic supplementary material for a general result. Let us stress that bi-modality in (2.5) is achieved by exploiting the stochasticity and discreteness of the controlling species *Y*_1_ and *Y*_2_; see [25] for more details on this noise-induced mixing phenomenon. In particular, the output networks (2.1) ∪ (2.4) displays a drastically different behaviour at the deterministic level (reaction-rate equations [49]): as a consequence of the deterministic dynamics and continuous abundances, the target species *X* is uni-stable with the unique equilibrium approaching zero as *ɛ* → 0.

PMF (2.5) is a linear combination of two Poisson distributions (bases), whose modes are controlled with the rate coefficients ***γ*** from the interfacing network $R_{\gamma }^{P}(X;\;Y_{1},Y_{2})$, while the PMF values at the modes (weights in (2.5)) are controlled with the rate coefficients ***β*** from the controller core $R_{\beta }(Y_{1},Y_{2})$. In particular, PMF (2.5) is independent of the asymptotic parameter *ɛ*, and depends on *β*_1,2_ and *β*_2,1_ only via the ratio *β*_1,2_/*β*_2,1_, which determines the PMF values at the two modes, which in turn depend on the ratios *γ*_1_/*γ*_0_ and *γ*_2_/*γ*_0_. However, the underlying sample paths do depend on *ɛ*, which determines the timescale of the fluctuations near each of the two modes, and on the parameters *β*_1,2_ and *β*_2,1_, which influence the sample paths independently and not only via their ratio. In particular, for a fixed ratio *β*_1,2_/*β*_2,1_, the value of *β*_1,2_ determines the timescale of stochastic switching between the two modes: given the system has started near *γ*_1_/*γ*_0_, it deterministically moves to a neighbourhood of *γ*_2_/*γ*_0_ after a random holding time which is exponentially distributed with mean 1/*β*_1,2_. These observations are instances of the fact that PMFs do not uniquely capture time parametrizations of the underlying sample paths, which we exploit for gaining strong control. Given a fixed ratio *β*_1,2_/*β*_2,1_, the precise values of the coefficients can be fixed with the constraint *β*_1,2_ + *β*_2,1_ = *c* > 0; unless otherwise stated, we set *c* = 1.

Let us set the two target modes for networks (2.1) ∪ (2.4) to *x* = *γ*_1_/*γ*_0_ = 5 and *x* = *γ*_2_/*γ*_0_ = 30, which is achieved by choosing e.g. ***γ*** = (*γ*_0_, *γ*_1_, *γ*_2_) = (1, 5, 30). In figure 2*c*, we display the corresponding long-time *x*-marginal PMFs for different values of *ɛ*, with (*β*_1,1_, ***β***) = (*β*_1,1_, *β*_1,2_, *β*_2,1_) = (1, 1/2, 1/2) chosen so that the two Poisson distributions from (2.5) have equal weights. One can notice that the uni-modal input PMF is morphed close to the bi-modal form (2.5), shown as the cyan histogram in figure 2*c*. A corresponding representative sample path is shown in the top sub-panel of figure 2*d*, with the mean time spent near each of the two modes given by 1/*β*_1,2_ = 1/*β*_2,1_ = 2 time-units. In the bottom sub-panel of figure 2*d*, we display a sample path for (*β*_1,1_, *β*_1,2_, *β*_2,1_) = (1, 1, 1), which also corresponds to the PMF shown as the histogram in figure 2*c*, but whose mean switching time is halved, 1/*β*_1,2_ = 1/*β*_2,1_ = 1. More generally, instead of balancing the two Poisson PMFs by choosing *β*_1,2_/*β*_2,1_ = 1, one can control the weights of each of the two Poisson PMFs from (2.5) in a number of ways. For example, in figure 2*e*,*f*, we set $\beta_{1,2}/\beta_{2,1}=2P(\gamma_{1}/\gamma_{0};\;\gamma_{1}/\gamma_{0})/P(\gamma_{2}/\gamma_{0};\;\gamma_{2}/\gamma_{0})\approx 5$, ensuring that the long-time PMF at the mode *x* = *γ*_2_/*γ*_0_ = 30 is approximately two times higher than at the mode *x* = *γ*_1_/*γ*_0_ = 5, which can be achieved by taking (*β*_1,1_, *β*_1,2_, *β*_2,1_) = (1, 5/6, 1/6).

#### 2.1.3. Tri-modality

Stochastic morpher can be utilized to achieve multi-modality beyond bi-modality at the PMF level, and a controlled switching pattern at the underlying sample path level. For example, let us morph the PMF of the input network (2.1) into a tri-modal one, with the modes *x* ∈ {5, 30, 55}. Furthermore, let the underlying sample paths spend on average 3 time-units in the neighbourhood of each of the modes, with the switching order 5 → 55 → 30, i.e. after being close to the mode *x* = 5, the system should jump near *x* = 55, then close to *x* = 30, before finally returning back to *x* = 5. To this end, consider the controller $R_{\beta }(Y_{1},Y_{2},Y_{3})\cup R_{\gamma }^{P}(X;\;Y_{1},Y_{2},Y_{3})$, given by

2.6 $$\begin{array}{rl} R_{\beta }\;:\; & 2Y_{1}\overset{\beta_{1,1}}{⟶}Y_{1}\overset{\beta_{1,2}}{⟶}Y_{2}\overset{\beta_{2,3}}{⟶}Y_{3}\overset{\beta_{3,1}}{⟶}Y_{1},\\ R_{\gamma }^{P}\;: & \;R_{\gamma_{0}}^{\varepsilon }\;:\;X\overset{\gamma_{0}/\varepsilon }{⟶}\text{Ø},\\ \;R_{\gamma_{1}}^{\varepsilon }\;: & \;Y_{1}\overset{\gamma_{1}/\varepsilon }{⟶}Y_{1}+X,\\ \;R_{\gamma_{2}}^{\varepsilon }\;: & \;Y_{2}\overset{\gamma_{2}/\varepsilon }{⟶}Y_{2}+X,\\ \;R_{\gamma_{3}}^{\varepsilon }\;: & \;Y_{3}\overset{\gamma_{3}/\varepsilon }{⟶}Y_{3}+X. \end{array}$$

Analogous to figure 2*a*–*f*, in figure 2*g*,*h* we display the long-time *x*-marginal PMF, and a representative sample path, of the output networks (2.1) ∪ (2.6), with ***γ*** = (*γ*_0_, *γ*_1_, *γ*_2_, *γ*_3_) = (1, 5, 55, 30) and (*β*_1,1_, *β*_1,2_, *β*_2,3_, *β*_3,1_) = (1, 1/3, 1/3, 1/3). In the asymptotic limit *ɛ* → 0, the PMF approaches the linear combination of three Poisson distributions centred at *x* = *γ*_1_/*γ*_0_ = 5, *x* = *γ*_2_/*γ*_0_ = 55 and *x* = *γ*_3_/*γ*_0_ = 30, each with equal weights (see theorem S5.1 in section S5 in the electronic supplementary material), which is in excellent agreement with the histogram from figure 2*g*, where parameter *ɛ* is two orders of magnitude larger than the rate coefficients from $R_{\alpha }^{1}(X)$ and $R_{\beta }(Y_{1},Y_{2},Y_{3})$. Note that the switching order of the sample path from figure 2*h* mirrors the periodic conversion *Y*_1_ → *Y*_2_ → *Y*_3_ → *Y*_1_ from (2.6). Let us stress that, despite having drastically different shapes and functionalities, the uni-modal and tri-modal PMFs displayed in figure 2*a*,*g*, respectively, have identical limiting mean (first moment) given by 30, which we show as red dashed lines in figure 2*b*,*h*. From the perspective of controllers that can manipulate only the first moment, control achieved in figure 2*a*,*g* is indistinguishable. On the other hand, using the stochastic morpher, a significantly finer control can be exerted by manipulating the higher-order moments and, thus, achieving greater biochemical functionality.

### 2.2. Higher-resolution control

In §2.1, we have applied the lower-resolution control consisting of the networks $R_{\beta }$ and $R_{\gamma }^{P}$, given generally by (S13) and (S14) in section S3 in the electronic supplementary material, respectively. In this section, we replace the uni-molecular lower-resolution (Poisson distribution) interfacing network $R_{\gamma }^{P}$ with its bi-molecular higher-resolution (Kronecker-delta distribution) counterpart $R_{\gamma }^{\delta }$, given by (S15) in section S3 in the electronic supplementary material.

#### 2.2.1. Kronecker-delta distribution

Consider the higher-resolution stochastic morpher $R_{\beta }\cup R_{\gamma }^{\delta }=R_{\beta }(Y_{1})\cup R_{\gamma }^{\delta }(X,Z_{1},Z_{2};\;Y_{1})$, given by

2.7 $$\begin{array}{rl} R_{\beta }\;: & \;2Y_{1}\overset{\beta_{1,1}}{⟶}Y_{1},\\ R_{\gamma }^{\delta }\;: & \;R_{\gamma_{0}}^{\mu ,\varepsilon ,\sigma }\;:\;\text{Ø}\overset{1/\varepsilon }{⟶}X,\\ & \;X\overset{\gamma_{0,1}}{\underset{1/\mu }{⇌}}Z_{1},\\ & \;X+Z_{1}\overset{\gamma_{0,2}}{\underset{1/\mu }{⇌}}Z_{2},\\ & \;R_{\gamma_{1}}^{\mu ,\varepsilon ,\sigma }\;:\;Y_{1}+Z_{2}\overset{\gamma_{1}}{⟶}Y_{1}+Z_{1}. \end{array}$$

Interfacing network $R_{\gamma }^{\delta }=R_{\gamma_{0}}^{\mu ,\varepsilon ,\sigma }\cup R_{\gamma_{1}}^{\mu ,\varepsilon ,\sigma }$ consists of two sub-networks: $R_{\gamma_{0}}^{\mu ,\varepsilon ,\sigma }$ describes a production of *X*, a reversible conversion of *X* into a controlling species *Z*_1_, and a reversible conversion of *X* and *Z*_1_ into another controlling species *Z*_2_. On the other hand, $R_{\gamma_{1}}^{\mu ,\varepsilon ,\sigma }$ describes an irreversible conversion of *Z*_2_ into *Z*_1_, catalysed by *Y*_1_. Under suitable conditions on the rate coefficients (in particular, with *μ*^2^*γ*_0,1_*γ*_0,2_*γ*_1_ = (*σɛ*)^−1^ and 0 < *μ* ≪ *ɛ*, *σ* ≪ 1), ensuring that the controlling species $Z=\{Z_{1},Z_{2}\}$ are sufficiently fast, network $R_{\gamma }^{\delta }$ from (2.7) reduces to

2.8 $$\begin{array}{rl} \;R_{\gamma_{0}}^{\varepsilon }\;:\; & \text{Ø}\overset{1/\varepsilon }{⟶}X,\\ \;R_{\gamma_{1}}^{\varepsilon ,\sigma }\;:\; & Y_{1}+2X\overset{1/(\sigma \varepsilon )}{\to }Y_{1}+X,\;\mathrm{as}\;\mu \to 0, \end{array}$$

see theorem S4.1 in section S4 in the electronic supplementary material. The first reaction from (2.8) generates a strong positive drift, which is overpowered by an even stronger negative drift induced by the second reaction when *x* ≥ 2. As a consequence, the long-time PMF of *X* from (2.1) ∪ (2.7) is concentrated at *x* = 1, and approaches the Kronecker-delta distribution centred at *x* = 1, see theorem S5.2 in section S5 in the electronic supplementary material. In figure 3*a*, we display the long-time *x*-marginal PMF of the output networks (2.1) ∪ (2.7), with the cyan histogram being in an excellent agreement with the Kronecker-delta distribution.

#### 2.2.2. Uniform distribution

Network (2.7) achieves a single Kronecker-delta distribution centred at *x* = 1. By introducing additional controlling species Y and Z, any number of Kronecker-delta distributions, centred at arbitrary points, are achievable. Using this approach, one can systematically morph a given input probability distribution into an arbitrary output one on a bounded domain, see section S5 for proof in the electronic supplementary material. For example, by placing three Kronecker-delta distributions at *x* ∈ {1, 2, 3} with equal weights, one obtains the uniform distribution on *x* ∈ {1, 2, 3}, see example S5.1 in section S5 in the electronic supplementary material and figure 3*b*.

#### 2.2.3. Hybrid control

The lower- and higher-resolution networks $R_{\gamma }^{P}$ and $R_{\gamma }^{\delta }$ from section S3 in the electronic supplementary material, respectively, can be combined into a composite hybrid scheme for biochemical control. For example, one may wish to obtain a more-detailed control over regions of the state-space where the target species are in lower copy numbers, while a less-detailed control may be sought over the higher copy-numbers state space. Such a hybrid approach may be experimentally desirable, as biochemical realizations of the Kronecker-delta PMFs centred at lower copy-numbers are less expensive to engineer, since a smaller number of the controlling species Z is required. For example, in figure 3*c*, we display morphing of the input PMF into a mixture of the Kronecker-delta distribution at *x* = 1 and the Poisson distribution at *x* = 30 via the controller (S57) from section S5 in the electronic supplementary material, while figure 3*d* shows a corresponding sample path.

### 2.3. Remarks about the stochastic morpher

The lower-resolution stochastic morpher is a negative-feedback controller; in particular, it introduces degradation reactions, and hence negative self-feedback loops, involving the target species $X_{\tau }$. The degradation reactions, such as X→Ø in (2.2), can be seen as approximations of *Y*_0_ + *X* → *Y*_0_, where *Y*_0_ is an auxiliary exclusive catalyst, or ${\bar{Y}}_{0}+X\to \text{Ø}$, where ${\bar{Y}}_{0}$ is a higher-concentration buffer. Alternatively, one can replace X→Ø with *Y*_1_ + *X* → *Y*_1_, without changing the conclusions made in this section, but at a possible experimental cost, see §5. Analogous conclusions hold for the zero-order production reactions in the higher-resolution controller. Note that the higher-resolution controller contains both negative and positive feedback loops involving the species $X_{\tau }$ and Z. Let us also note that we require the total initial copy number of the controlling species to be non-zero; such a condition is unavoidable, as any network, once implemented, depends on the presence of suitable (buffer) species.

## 3. Bi-stable input network

In this section, we apply stochastic morpher to a more complicated reaction network, highlighting how multiple target species can be jointly controlled. To this end, consider the three-species bi-molecular input network $R_{\alpha }^{2}=R_{\alpha }^{2}(X_{1},X_{2},X_{3})$, given by

3.1 $$\begin{array}{rl} R_{\alpha }^{2}\;: & \;\text{Ø}\overset{\alpha_{1}}{\underset{\alpha_{2}}{⇌}}X_{1},\;\text{Ø}\overset{\alpha_{3}}{⟶}X_{2},\;\text{Ø}\overset{\alpha_{4}}{⟶}X_{3},\;X_{3}\overset{\alpha_{5}}{⟶}X_{1},\\ & \;2X_{1}\overset{\alpha_{6}}{⟶}2X_{1}+X_{3},\;X_{1}+X_{2}\overset{\alpha_{7}}{⟶}2X_{1},\;X_{1}+X_{3}\overset{\alpha_{8}}{⟶}X_{3}. \end{array}$$

**Figure 4.**
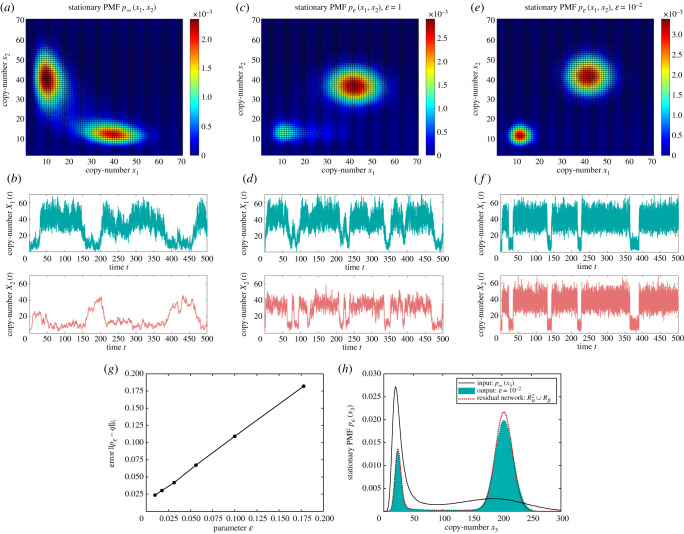
Application of the lower-resolution stochastic morpher on the input network (3.1) with ***α*** = (*α*_1_, *α*_2_, *α*_3_, *α*_4_, *α*_5_, *α*_6_, *α*_7_, *α*_8_) = (2, 7/2, 2, 18, 3/2, 9/50, 1/200, 1/48). (*a*,*b*) The long-time (*x*_1_, *x*_2_)-marginal PMF of the input network (3.1), and the underlying representative sample paths for target species *X*_1_ and *X*_2_, respectively. Analogous plots are shown in (*c*,*d*) and (*e*,*f*) for the output network (3.1) ∪ (3.2) with ***β*** = (*β*_1,1_, *β*_1,2_, *β*_2,1_) = (1, 4/50, 1/50), ***γ*** = (***γ***_1_, ***γ***_2_) = ((*γ*_0,1_, *γ*_1,1_, *γ*_2,1_), (*γ*_0,2_, *γ*_1,2_, *γ*_2,2_)) = ((1, 10, 40), (1, 10, 40)), and different values of *ɛ*. (*g*) Displays as the black dots, interpolated with the black lines, a plot of the *l*^1^-distance between the target PMF (3.3) and the long-time PMF of output network (3.1) ∪ (3.2) as a function of *ɛ*. (*h*) The long-time *x*_3_-marginal PMFs of the input network (3.1), and the output network (3.1) ∪ (3.2) with *ɛ* = 10^−2^, as the black solid curve, and the cyan histogram, respectively. Also shown, as the dotted red curve, is the long-time *x*_3_-marginal PMFof the network (S64)$\;\cup \;R_{\beta }(Y_{1},Y_{2})$.

It is assumed that we can explicitly control the target species $X_{\tau }=\{X_{1},X_{2}\}$, with the remaining (residual) species being $X_{\rho }=\{X_{3}\}$. For a particular choice of the input rate coefficients ***α***, the long-time (*x*_1_, *x*_2_)-marginal PMF of network (3.1) is shown in figure 4*a*, while the underlying sample paths for *X*_1_ and *X*_2_ are shown in cyan and red in figure 4*b*, respectively. The (*x*_1_, *x*_2_)-marginal PMF is bi-modal, with the approximate modes (*x*_1_, *x*_2_) = (10, 40) and (*x*_1_, *x*_2_) = (40, 10), and the species *X*_1_ and *X*_2_ are negatively correlated.

In what follows, we apply the stochastic morpher in order to reshape the input PMF from figure 4*a* into a bi-modal one, with the species *X*_1_ and *X*_2_ being positively correlated; in context of cellular control, changing the sign of the correlation between some of the underlying regulatory proteins can give rise to different cell phenotypes with new survival strategies [46]. More precisely, let us morph the input modes into the target modes given by (*x*_1_, *x*_2_) = (10, 10) and (*x*_1_, *x*_2_) = (40, 40), where the (*x*_1_, *x*_2_)-marginal PMF takes approximately the same values, and with the switching time between the two new modes being of the order O(10) time-units. To this end, consider the stochastic morpher $R_{\beta }\cup R_{\gamma }^{P}=R_{\beta }(Y_{1},Y_{2})\cup R_{\gamma }^{P}(X_{1},X_{2};\;Y_{1},Y_{2})$, given by

3.2 $$\begin{array}{rl} R_{\beta }\;: & \;2Y_{1}\overset{\beta_{1,1}}{⟶}Y_{1}\overset{\beta_{1,2}}{\underset{\beta_{2,1}}{⇌}}Y_{2},\\ R_{\gamma }^{P}\;: & \;R_{\gamma_{0}}^{\varepsilon }\;:\;X_{1}\overset{\gamma_{0,1}/\varepsilon }{⟶}\text{Ø},\;X_{2}\overset{\gamma_{0,2}/\varepsilon }{⟶}\text{Ø},\\ \;R_{\gamma_{1}}^{\varepsilon }\;: & \;Y_{1}\overset{\gamma_{1,1}/\varepsilon }{⟶}Y_{1}+X_{1},\;Y_{1}\overset{\gamma_{1,2}/\varepsilon }{⟶}Y_{1}+X_{2},\\ \;R_{\gamma_{2}}^{\varepsilon }\;: & \;Y_{2}\overset{\gamma_{2,1}/\varepsilon }{⟶}Y_{2}+X_{1},\;Y_{2}\overset{\gamma_{2,2}/\varepsilon }{⟶}Y_{2}+X_{2}, \end{array}$$

which is a two-species analogue of the network (2.4). As *ɛ* → 0, the long-time (*x*_1_, *x*_2_)-marginal PMF of the output networks (3.1)∪(3.2) approaches (see theorem S5.1 in section S5 in the electronic supplementary material)

3.3 $$\begin{array}{rl} q(x_{1},x_{2})= & {\left(1+\frac{\beta_{1,2}}{\beta_{2,1}}\right)}^{-1}P\left(x_{1};\;\frac{\gamma_{1,1}}{\gamma_{0,1}}\right)P\left(x_{2};\;\frac{\gamma_{1,2}}{\gamma_{0,2}}\right)\\ & +{\left(1+\frac{\beta_{2,1}}{\beta_{1,2}}\right)}^{-1}P\left(x_{1};\;\frac{\gamma_{2,1}}{\gamma_{0,1}}\right)P\left(x_{2};\;\frac{\gamma_{2,2}}{\gamma_{0,2}}\right). \end{array}$$

In order to achieve the desired modes, we fix ***γ*** = (***γ***_1_, ***γ***_2_) = ((*γ*_0,1_, *γ*_1,1_, *γ*_2,1_), (*γ*_0,2_, *γ*_1,2_, *γ*_2,2_)) = ((1, 10, 40), (1, 10, 40)). On the other hand, in order to ensure that the PMF takes approximately equal values at the two modes and that the switching time is of the order O(10) time units, we set *β*_1,2_/*β*_2,1_ = 4 and (*β*_1,2_ + *β*_2,1_) = 1/10, respectively, which is achieved by taking (*β*_1,1_, *β*_1,2_, *β*_2,1_) = (1, 4/50, 1/50). In figure 4*c*,*d*, we display the long-time (*x*_1_, *x*_2_)-marginal PMF of networks (3.1) ∪ (3.2), and the underlying representative sample paths, when the asymptotic parameter is fixed to *ɛ* = 1, showing that the desired bi-modality and positive correlation between *X*_1_ and *X*_2_ are already achieved. Note that the input PMF is at first more attracted towards the target mode containing more probability mass as *ɛ* → 0, which, under the particular choice of ***β***, is the mode (*x*_1_, *x*_2_) = (40, 40). In figure 4*e*,*f*, we set *ɛ* = 10^−2^, and one can notice an excellent match with the asymptotic prediction (3.3).

To gain more quantitative information about the convergence, in figure 4*g* we display the distance (error) between the long-time PMF of the output networks (3.1) ∪ (3.2), denoted by *p*_*ɛ*_(*x*_1_, *x*_2_), and the target PMF (3.3), as a function of the asymptotic parameter *ɛ*. Measuring the error with the *l*^1^-norm, $∥p_{\varepsilon }-q{∥}_{l_{1}}=\sum_{x_{1},x_{2}}|p_{\varepsilon }(x_{1},x_{2})-q(x_{1},x_{2})|$, one can notice that $∥p_{\varepsilon }-q{∥}_{l_{1}}=O(\varepsilon )$ for sufficiently small *ɛ*. In fact, more generally, assuming suitable stability of the output network, convergence of the time-dependent output PMF to the target PMF is exponential in time *t* and linear in parameter *ɛ*, see theorem S5.1 in section S5 in the electronic supplementary material.

Note that the explicit control of the target species $X_{\tau }=\{X_{1},X_{2}\}$ implicitly influences the residual species $X_{\rho }=\{X_{3}\}$. We demonstrate this influence in figure 4(h), where the long-time *x*_3_-marginal PMFs from the input network (3.1) is displayed as the solid black curve, while from the output networks (3.1) ∪ (3.2) with *ɛ* = 10^−2^ as the cyan histogram, showing that the PMF is redistributed across two approximately fixed modes. Also shown, as the dotted red curve, is a prediction of the *x*_3_-marginal PMF in the limit *ɛ* → 0, based on the results from section S6 in the electronic supplementary material; in particular, see theorem S6.1 and example S6.1 in the electronic supplementary material.

## 4. Implicit control: gene expression input network

In §3, we focused on explicitly controlling the target species, while ignoring the underlying residual effects. In this section, we shift our focus to an implicit control of the residual species via appropriate explicit manipulation of the target species. In particular, we show that such an implicit control can be achieved via the lower-resolution stochastic morpher by sufficiently slowing down the controller core $R_{\beta }$, in addition to sufficiently speeding up the controller interface $R_{\gamma }$. To this end, consider the two-species uni-molecular reaction network $R_{\alpha }^{3}=R_{\alpha }^{3}(X_{1},X_{2})$, given by

4.1 $$R_{\alpha }^{3}\;:\;\text{Ø}\overset{\alpha_{1}}{\underset{\alpha_{2}}{⇌}}X_{1},\;X_{1}\overset{\alpha_{3}}{⟶}X_{1}+X_{2},\;X_{2}\overset{\alpha_{4}}{⟶}\text{Ø}.$$

**Figure 5.**
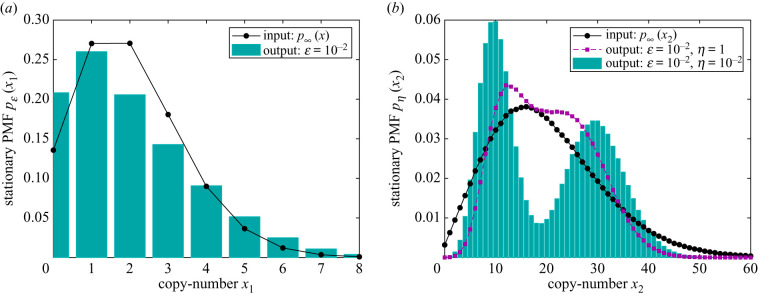
Application of the lower-resolution stochastic morpher on the input network (4.1) with (*α*_1_, *α*_2_, *α*_3_, *α*_4_) = (2, 1, 10, 1). (*a*) The long-time *x*_1_-marginal PMF of the input network (4.1) as the interpolated black dots, and of the output network (4.1) ∪ (4.2), with (*β*_1,1_, *β*_1,2_, *β*_2,1_) = (1, 1/2, 1/2), (*γ*_0,1_, *γ*_1,1_, *γ*_2,1_) = (1, 1, 3) and *η* = 1, as the cyan histogram. (*b*) The input long-time *x*_2_-marginal PMF as the interpolated black dots. Also shown, under the same rate coefficients, is the long-time *x*_2_-marginal PMF of (4.1) ∪ (4.2) for different values of *η*.

Network (4.1) can be seen as an extension of (2.1), describing a simplified model for an intracellular gene expression [37]: *X*_1_ represents an mRNA, transcribed from a gene and translated into a protein *X*_2_, with each of the two species being degradable. In figure 5*a*,*b*, we display the uni-modal stationary PMFs of the target and residual species *X*_1_ and *X*_2_ from (4.1), respectively, for a particular choice of the input coefficients ***α***. The goal in this section is to implicitly induce bi-modality into the protein (residual) species *X*_2_ (and thereby create cells with two phenotypes) by explicitly influencing the mRNA (target) species *X*_1_. Such a setting is suitable when RNA-based controllers are utilized which, due to their biophysical properties, are generally more readily interfaced with mRNAs than with proteins [7,17].

As opposed to explicit control, considered in §§2 and 3, where black-box input networks have been considered, implicit control can be applied only to grey-box networks, as one requires at least partial knowledge about the underlying structure and dynamics, see also section S6 in the electronic supplementary material. In what follows, we assume that the reactions which change the copy-number of *X*_2_ are known and given by *X*_1_ → *X*_1_ + *X*_2_ and $X_{2}\to \text{Ø}$; we allow the underlying rate coefficients (*α*_3_, *α*_4_) to remain unknown. To implicitly induce bi-modality into the PMF of *X*_2_, let us consider the stochastic morpher $R_{\beta }^{\eta }\cup R_{\gamma }^{P}=R_{\beta }^{\eta }(Y_{1},Y_{2})\cup R_{\gamma }^{P}(X_{1};\;Y_{1},Y_{2})$, given by

4.2 $$\begin{array}{rl} R_{\beta }^{\eta }\;: & \;2Y_{1}\overset{\beta_{1,1}}{⟶}Y_{1}\overset{\eta \beta_{1,2}}{\underset{\eta \beta_{2,1}}{⇌}}Y_{2},\\ R_{\gamma }^{P}\;: & \;R_{\gamma_{0}}^{\varepsilon }\;:\;X_{1}\overset{\gamma_{0,1}/\varepsilon }{⟶}\text{Ø},\\ \;R_{\gamma_{1}}^{\varepsilon }\;: & \;Y_{1}\overset{\gamma_{1,1}/\varepsilon }{⟶}Y_{1}+X_{1},\\ \;R_{\gamma_{2}}^{\varepsilon }\;: & \;Y_{2}\overset{\gamma_{2,1}/\varepsilon }{⟶}Y_{2}+X_{1}. \end{array}$$

In the limit *ɛ* → 0, the effective behaviour of species *X*_2_ is described by the so-called *residual network* ${\bar{R}}_{\alpha }^{3}={\bar{R}}_{\alpha }^{3}(X_{2};\;Y_{1},Y_{2})$, obtained by averaging out the equilibrated target species *X*_1_, and given by

4.3 $$\begin{array}{rl} {\bar{R}}_{\alpha }^{3}\;: & \;X_{2}\overset{\alpha_{4}}{⟶}\text{Ø},\\ & \;Y_{1}\overset{\alpha_{3}\left(\frac{\gamma_{1,1}}{\gamma_{0,1}}\right)}{\to }Y_{1}+X_{2},\;Y_{2}\overset{\alpha_{3}\left(\frac{\gamma_{2,1}}{\gamma_{0,1}}\right)}{\to }Y_{2}+X_{2},\;\mathrm{as}\;\varepsilon \to 0, \end{array}$$

see theorem S6.1 in section S6 in the electronic supplementary material. By further letting *η* → 0 in (4.2), one finds that the *x*_2_-marginal PMF converges close to

4.4 $$\begin{array}{rl} Q(x_{2}) & ={\left(1+\frac{\beta_{1,2}}{\beta_{2,1}}\right)}^{-1}P\left(x_{2};\;\frac{\alpha_{3}}{\alpha_{4}}\left(\frac{\gamma_{1,1}}{\gamma_{0,1}}\right)\right)\\ & \;+{\left(1+\frac{\beta_{2,1}}{\beta_{1,2}}\right)}^{-1}P\left(x_{2};\;\frac{\alpha_{3}}{\alpha_{4}}\left(\frac{\gamma_{2,1}}{\gamma_{0,1}}\right)\right), \end{array}$$

see theorem S6.2 in section S6 in the electronic supplementary material. In contrast to the explicitly achieved PMFs in §§2 and 3, the implicitly achieved PMF (4.4) is not robust, as its modes depend explicitly on the input rate coefficients *α*_3_ and *α*_4_; however, note that the weights in (4.4) are robust. Furthermore, for a fixed ratio (*β*_1,2_/*β*_2,1_), the free parameter (*β*_1,2_ + *β*_2,1_) is utilized in §§2 and 3 to achieve strong control of the underlying sample paths; in this section, we utilize this degree of freedom to sufficiently slow down the network $Y_{1}⇌Y_{2}$, i.e. we take *η*(*β*_1,2_ + *β*_2,1_) ≪ 1 to achieve implicit weak control.

While the modes from (4.4) are not robust, their ratio, (*γ*_2,1_/*γ*_1,1_), is robust, see corollary S6.1 in section S6 in the electronic supplementary material for a more general result. Such robustness is inherited from the target species; in particular, the ratio between the modes of the implicitly controlled species (protein) is identical to the ratio between the modes of the explicitly controlled species (mRNA). Taking (*γ*_2,1_/*γ*_1,1_) sufficiently large ensures that the two Poisson distributions from (4.4) are well-separated, and bi-modality achieved. Assume we want the larger mode to be three times further away than the smaller mode, with the two Poisson distributions having equal weights. Such constraints are achievable by the output networks (4.1) ∪ (4.2) with ***γ*** = (*γ*_0,1_, *γ*_1,1_, *γ*_2,1_) = (1, 1, 3) and (*β*_1,1_, *ηβ*_1,2_, *ηβ*_2,1_) = (1, *η*/2, *η*/2), with 0 < *η* ≪ 1. In figure 5*a*, the cyan histogram displays the long-time *x*_1_-marginal PMF of (4.1)∪(4.2) when *ɛ* = 10^−2^. On the other hand, in figure 5*b*, we display the long-time *x*_2_-marginal PMF of the output networks (4.1) ∪ (4.2) with *ɛ* = 10^−2^ for two different values of *η*, and one can notice convergence close to the target (4.4). Note that the output *x*_1_-marginal PMF is uni-modal, as the underlying two Poisson distributions are not well-separated; on the other hand, the limiting *x*_2_-marginal PMF is bi-modal, as the modes are magnified by a factor of *α*_3_/*α*_4_ = 10. Let us also note that, if the ratio *α*_3_/*α*_4_ is experimentally measured for the grey-box input network (4.1), then each of the protein modes, and not only their ratio, can be implicitly controlled; in fact, *α*_3_/*α*_4_ can be deduced from the action of the stochastic morpher.

## 5. Proposed experimental implementation: synthetic cells

In this section, we put forward a blueprint for an *in vitro* experimental implementation of stochastic morpher via dynamic nucleic acid nanotechnology based on strand-displacement reactions. Structurally, stochastic morpher involves up to bi-molecular reactions, some of which are catalytic (non-elementary), while its dynamical operation depends on time-separated kinetics. DNA/RNA strand-displacement can in principle meet both of these requirements; in particular, up to bi-molecular reactions, with arbitrary product and reactant species compositions, including catalytic reactions, are readily mapped to elementary reactions when compiled into nucleic-acid-based physical networks [14,51–53]. On the other hand, the rate coefficients can be varied over at least six orders of magnitude under strand displacement [16,17,54], allowing for timescale separations. The lower-resolution control, consisting of the networks (S13)–(S14) in section S3 in the electronic supplementary material, is biochemically less costly, as it involves only two timescales and only one bi-molecular reaction; its higher-resolution counterpart, given by (S13)–(S15), is biochemically more costly, since it contains a larger number of timescales, bi-molecular reactions and auxiliary species. Let us stress that quasi-robust control can be achieved with stochastic morpher by tuning ratios and orders of magnitude of the underlying rate coefficients, and not their precise values, as outlined in §§2–4, which is a task achievable within strand-displacement DNA/RNA computing. Furthermore, stochastic morpher transforms PMFs through a sequence of intermediate probability distributions that increasingly resemble the desired target distribution; therefore, satisfactory partial control may be achievable even under weaker timescale separations, e.g. see the purple intermediate PMFs from figure 2, which already display some desirable properties.

### 5.1. DNA Holliday junction and vesicle encapsulation

**Figure 6.**
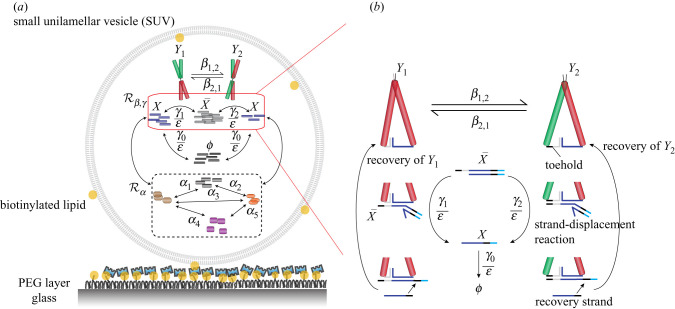
Proposed experimental scheme for the stochastic morpher (2.4). (*a*) Displays an SUV immobilized onto a PEG or BSA passivated surface, containing an input network $R_{\alpha }$, enclosed in a dashed rounded rectangle, which is coupled to the controller $R_{\beta ,\gamma }$, included in the red rounded rectangle. The controller includes a single DNA Holliday junction molecule, which switches between two distinct orientations, denoted by *Y*_1_ and *Y*_2_, and catalytically produces the target species *X*, which is then degraded by suitable strand-displacement reactions. (*b*) Displays in a greater detail the underlying strand-displacement reactions that produce *X*, and which are enclosed in the red rounded rectangle in (*a*). The single-stranded DNA overhangs on the Holliday junction associate pairwise, forming a toehold and a branch-migration domain, shown in grey/black and blue on *Y*_1_ and *Y*_2_, respectively, which are separated by a duplex. The association-activated toeholds bind to an auxiliary double-stranded DNA species $\bar{X}$, initiating the release of the target molecule *X*; *Y*_1_ and *Y*_2_ are then recovered via strand-displacement reactions initiated by a suitable recovery strand.

As a proof-of-concept, we propose an experimental realization of the lower-resolution stochastic morpher (2.4) from §2, which is capable of achieving bi-modality. One way to realize (2.4) is via a biochemical network satisfying the following two properties: (i) it contains two isomeric molecular species which can inter-convert (realizing the controlling species *Y*_1_ and *Y*_2_, and the reaction $Y_{1}⇌Y_{2}$), each triggering a catalytic production of the target species *X* at generally different rates (realizing the reactions *Y*_1_ → *Y*_1_ + *X* and *Y*_2_ → *Y*_2_ + *X*), and (ii) the network is integrated into an environment with exactly one copy number of the two isomeric species (realizing the conservation law (*y*_1_ + *y*_2_) = 1, and eliminating the reaction 2*Y*_1_ → *Y*_1_). We propose to achieve these conditions using a DNA complex, known as the Holliday junction molecule, encapsulated in a nano-scale compartment, known as the small unilamellar vesicle (SUV) [9–12,55], forming a bi-phenotypic synthetic cell schematically displayed in figure 6*a*. The Holliday junction consists of four double-stranded arms crossing at a branch point, designed to be fixed (non-migratory) for our purposes. In the presence of magnesium ions, the Holliday junction can adopt two distinct orientations, known as stacked conformational isomers [19,56], realizing the reversible reaction $Y_{1}⇌Y_{2}$. On the other hand, the SUV encapsulation is an experimentally demonstrated method for isolating and observing the dynamics of individual molecules with minimal effect from the external environment [12]. Under a sufficiently low concentration of *Y*_1_ and *Y*_2_ during vesicle assembly, one can utilize single-molecule fluorescence [57] to identify SUVs containing exactly one copy number of the Holliday junction.

### 5.2. Strand-displacement reactions

In order to control the rate coefficients of the reactions $Y_{1}⇌Y_{2}$, *Y*_1_ → *Y*_1_ + *X* and *Y*_2_ → *Y*_2_ + *X*, we put forward suitable DNA overhangs involved in the associative toehold activation [58]. More precisely, we propose to extend (tag) three arms of the four-armed Holliday junction with distinct single-stranded DNA molecules (overhangs), which can associate in a pairwise manner, thereby activating distinct toeholds shown as the grey/black regions attached to *Y*_1_ and *Y*_2_ in figure 6*b*. Furthermore, the overhangs also partially hybridize with each other, forming duplexes next to the toeholds. By controlling the length, and therefore the binding energy, of the duplexes in the associated DNA overhangs, one can experimentally tune the kinetics of reaction $Y_{1}⇌Y_{2}$. On the other hand, by controlling the length of the association-activated toeholds, one can independently tune the rates of *Y*_1_ → *Y*_1_ + *X* and *Y*_2_ → *Y*_2_ + *X*, by influencing the two subsequent strand-displacement reactions which produce the target species *X* from a suitable precursor molecule, denoted by $\bar{X}$ in figure 6*b*. Once a molecule of *X* is produced, *Y*_1_ and *Y*_2_ can be recovered via strand-displacement reactions initiated by a suitable single-stranded DNA, called a recovery strand, ensuring an effective catalytic role of *Y*_1_ and *Y*_2_ in the overall reaction cascade. Let us note that, despite a similarity in the domains of *X* and the recovery strand, the two strands have distinct toeholds, which we show in light blue and black in figure 6*b*, respectively; therefore, *X* and the recovery strand are biochemically distinct. Hence, one can design downstream strand-displacement reactions that would be activated by the unique toehold on *X* and subsequently monitored. For example, *X* could invade the desired DNA duplex labelled with a fluorophore and a quencher; the displaced fluorophore-tagged DNA strand can then be detected through the increase in fluorescence.

The operational timescale of the stochastic morpher is limited by the abundance of the DNA complexes that act as substrates for activation and degradation of *X*, and for recovery of *Y*_1_ and *Y*_2_. When a significant fraction of the resources is consumed, the underlying effective rate coefficients slow down and the action of the controller weakens. This time limitation can be relaxed by replenishing the resources either externally by using semi-permeable SUVs with porous membranes [59], or internally by encapsulating suitable DNA buffers [60]. In fact, the proposed gene-like implementation of reaction $Y_{1}⇌Y_{2}$, via Holliday junction molecules, does not require buffer species, and in principle has a longer operational time when compared to the DNA strand-displacement implementation of $Y_{1}⇌Y_{2}$ put forward in [14].

## 6. Discussion

In this paper, we have introduced a molecular controller (see section S2 in the electronic supplementary material and figure 1 for a general outline), called *stochastic morpher*, that can, under suitable conditions, gradually transform (morph) the PMF of a given black-box input network into a predefined shape. Two forms of the controller have been put forward: the *lower-resolution* controller, given by (S13)–(S14) in section S3 in the electronic supplementary material, that morphs input PMFs into linear combinations of Poisson distributions, and its *higher-resolution* counterpart, given by (S13)–(S15) in section S3 in the electronic supplementary material, that achieves linear combinations of Kronecker-delta distributions. The control can be accomplished *explicitly* on the target species, or *implicitly* on the residual species via the target species. We have showcased the capabilities of the stochastic morpher on particular biochemical networks in §§2–4. More broadly, we have established general properties of the stochastic morpher in section S4 in the electronic supplementary material using singular perturbation theory [61], and specialized the results to explicit and implicit control in sections S5 and S6 in the electronic supplementary material, respectively. More precisely, we have shown in theorems S5.1–S5.2 that, under the assumption that the output networks display a suitable form of stability, both lower- and higher-resolution stochastic morphers achieve the desired explicit control. In particular, the PMF of the explicitly controlled species converges exponentially fast in time to the desired target distribution, up to an error that decreases linearly in the asymptotic parameter *ɛ*. Importantly, the target distributions are independent of the input rate coefficients, and the convergence is robust to the initial conditions, implying long-time quasi-robustness of the stochastic morpher. In theorem S5.3, we have proved that the required stability condition, and hence the results from theorems S5.1–S5.2, hold for any first-order input network. Analogous properties have been established for a class of implicitly controlled residual networks in theorem S6.2. To the best of our knowledge, stochastic morpher is the first controller that can exert control beyond the mean and variance, being able to achieve any probability distribution; in particular, one can achieve desired multi-modal distributions (weak control) whose sample paths have controlled timing and mode-switching pattern (strong control). Furthermore, stochastic morpher can also accomplish implicit control of the residual species, i.e. control of biochemical species that do not directly interact with the controlling species; in contrast, the direct interaction is necessary in some other works, e.g. in the robust controller from [39].

The lower-resolution stochastic morpher incorporates simple production and degradation of the target species as its faster sub-network. Faster production and degradation processes are omnipresent in living cells, where the continuous turnover of RNA molecules and proteins naturally shields the cells against internally and externally induced fluctuations, and reduces the retro-active load placed on the molecular circuits [52]. In control theory context, rapid production and degradation processes are known as high-gain negative-feedback controllers [62]. A high-gain feedback controller has been previously put forward for destroying all but one stable equilibrium, in a given class of multi-stable gene-regulatory networks, thus achieving uni-stability [41,42]. In this paper, we have instead combined high-gain feedback control with noise-induced mixing to systematically morph probability distributions into any predefined shape, with a particular focus on multi-modality/multi-stability. On the other hand, the higher-resolution stochastic morpher shares some similarities with the work presented in [63], where Kronecker-delta distributions are also implemented using timescale separations and catalysis similar to [25,64]. However, while we have realized Kronecker-delta distributions with experimentally feasible bi-molecular networks, implementations from [63] involve higher than bi-molecular networks. In electronic supplementary material, section S4, using the results from [28], we have proved that the bi-molecular construction put forward in this paper reduces in an appropriate limit to the higher-molecular one from [63]. Furthermore, in this paper, we have focused on biochemical control, involving combining multiple biochemical networks together, some of which are black-box (unknown), in order to achieve suitable dynamical behaviours. Therefore, our analyses take into a consideration realistic effects biochemical networks experience in applications, such as retro-activity [52,65]; such effects have not been included in [63].

In §5, we have put forward a blueprint for an *in vitro* experimental realization of the bi-modal stochastic morpher (2.4) using a DNA-based biochemical network encapsulated inside a nano-scale vesicle, thus implementing a bi-phenotypic synthetic cell shown in figure 6. We have proposed to appropriately assemble the vesicles so that the reaction 2*Y*_1_ → *Y*_1_ from (2.4) can be eliminated. However, let us note that such a reaction can be achieved within the proposed set-up by replacing the Holliday junction molecules with suitable DNA dimers. In particular, we propose assembling sufficiently rigid dimers containing the Holliday junction and a corresponding complementary motif (deactivator) on the opposite end, see [66] for details on multimer assembly of DNA nanostructures. When multiple dimers are present, a sequence of associations between the dimers would take place, sequestering all but one active Holliday junction, thus implementing 2*Y*_1_ → *Y*_1_. Let us also stress that, in this paper and §5 in particular, we have assumed that the molecular abundances are spatially homogeneous (well-mixed) inside reactors at any given time. This assumption holds if the stochastic morpher is implemented in sufficiently small compartments. However, for larger compartments, such as living cells, spatial heterogeneity of molecular abundances can play important roles [67–71]. To address such challenges, further theoretical and experimental considerations are required, which are beyond the scope of this paper.

Neglecting spatial heterogeneity in the molecular abundances, the lower-resolution stochastic morpher can also in principle be implemented *in vivo* via synthetic RNA-based networks encoded genetically inside cells; for concreteness, we focus on the bi-modal case (2.4). We propose introducing a synthetic plasmid, coding for a target micro-RNA species, into an *Escherichia coli* cell. In this context, the slowly interconverting unit copy-number controlling species are realized via different states of a suitable plasmid gene, and the switching between the two states is regulated by the slower binding and unbinding of suitable transcription factors giving rise to different degrees of promoter activity. The production of the target RNA is achieved via transcription from the plasmid gene, with the maximum transcription rate of the order of 10^−1^ s^−1^ [72], and we propose exploiting the natural intracellular degradation of RNA molecules, occurring at a rate of around 3 × 10^−3^ s^−1^ [73]. These estimations imply that the stochastic morpher $R_{\beta ,\gamma }$ can achieve multi-modal distributions with modes of up to approximately 30 RNA molecules per cell. To ensure quasi-robustness of the controller $R_{\beta ,\gamma }$, we require that the desired input network $R_{\alpha }$ fires at a slower timescale. For the interactions between the RNA species at a concentration of 30 molecules per cell, 0.1 reactions per second corresponds to a rate constant of the order of $10^{6}\;M^{-1}\;s^{-1}$, whereas the speed limit of nucleic-acid reactions inside cells is of the order of $10^{8}\;M^{-1}\;s^{-1}$ [74]. If $R_{\alpha }$ is a synthetic RNA-based network, the quasi-robust limit can be achieved by slowing down the input network via sequestration of the key domains in the secondary structures of the underlying species [17,54]. On the other hand, if $R_{\alpha }$ is a native RNA-based network, the quasi-robust limit might be challenging to achieve. In this context, an approach for speeding up the stochastic morpher is by transcribing sufficiently long RNA chains that are broken down into multiple target RNA molecules post-transcription via suitable ribozymes [75].

## Data accessibility

Data supporting this paper are contained within the paper, and as the electronic electronic supplementary material.

## References

[1] Endy D. 2005 Foundations for engineering biology. Nature **484**, 449-453. (10.1038/nature04342) 16306983

[2] Del Vecchio D, Dy AJ, Qian Y. 2016 Control theory meets synthetic biology. J. R. Soc. Interface **13**, 3-43. (10.1098/rsif.2016.0380) PMC4971224 27440256

[3] Gardner TS, Cantor CR, Collins JJ. 2000 Construction of a genetic toggle switch in *Escherichia coli*. Nature **403**, 339-342. (10.1038/35002131) 10659857

[4] Elowitz MB, Leibler S. 2000 A synthetic oscillatory network of transcriptional regulators. Nature **403**, 335-338. (10.1038/35002125) 10659856

[5] Boo A, Ellis T, Stan GB. 2019 Host-aware synthetic biology. Curr. Opin. Syst. Biol. **14**, 66-72. (10.1016/j.coisb.2019.03.001)

[6] Chappell J, Takahashi MK, Lucks JB. 2015 Creating small transcription activating RNAs. Nat. Chem. Biol. **11**, 214-220. (10.1038/nchembio.1737) 25643173

[7] Isaacs FJ, Dwyer DJ, Ding C, Pervouchine DD, Cantor CR, Collins JJ. 2004 Engineered riboregulators enable post-transcriptional control of gene expression. Nat. Biotechnol. **22**, 841-847. PreTranscription, PostTranscription. (10.1038/nbt986) 15208640

[8] Hsiao V, de Los Santos ELC, Whitaker WR, Dueber JE, Murray RM. 2014 Design and implementation of a biomolecular concentration tracker. ACS Synth. Biol. **4**, 150-161. (10.1021/sb500024b) 24847683 PMC4384833

[9] Hasatani K, Leocmach M, Genot AJ, Estévez-Torres A, Fujii T, Rondelez Y. 2013 High-throughput and long-term observation of compartmentalized biochemical oscillators. ChemComm **49**, 8090-8092. (10.1039/c3cc44323j) 23912586

[10] Weitz M, Kim J, Kapsner K, Winfree E, Franco E, Simmel FC. 2014 Diversity in the dynamical behaviour of a compartmentalized programmable biochemical oscillator. Nat. Chem. **6**, 295-302. (10.1038/nchem.1869) 24651195

[11] Genot AJ, Baccouche A, Sieskind R, Aubert-Kato N, Bredèche N, Bartolo JF, Taly V, Fujii T, Rondelez Y. 2016 High-resolution mapping of bifurcations in nonlinear biochemical circuits. Nat. Chem. **8**, 760. (10.1038/nchem.2544) 27442281

[12] Okumus B, Wilson TJ, Lilley DMJ, Ha T. 2004 Vesicle encapsulation studies reveal that single molecule ribozyme heterogeneities are intrinsic. Biophys. J. **87**, 2798-2806. (10.1529/biophysj.104.045971) 15454471 PMC1304698

[13] Gópfrich K, Urban MJ, Frey C, Platzman I, Spatz JP, Liu N. 2020 Dynamic actuation of DNA-assembled plasmonic nanostructures in microfluidic cell-sized compartments. Nano Lett. **20**, 1571-1577. (10.1021/acs.nanolett.9b04217) 32083879 PMC7307956

[14] Soloveichik D, Seeing G, Winfree E. 2010 DNA as a universal substrate for chemical kinetics. Proc. Natl Acad. Sci. USA **107**, 5393-5398. (10.1073/pnas.0909380107) 20203007 PMC2851759

[15] Srinivas N, Parkin J, Seeing G, Winfree E, Soloveichik D. 2017 Enzyme-free nucleic acid dynamical systems. Science **358**, eaal2052. (10.1126/science.aal2052) 29242317

[16] Zhang DY, Winfree E. 2009 Control of DNA strand displacement kinetics using toehold exchange. J. Am. Chem. Soc. **131**, 17 303-17 314. (10.1021/ja906987s) 19894722

[17] Šulc P, Ouldridge TE, Romano F, Doye JPK, Louis AA. 2015 Modelling toehold-mediated RNA strand displacement. Biophys. J. **108**, 1238-1247. (10.1016/j.bpj.2015.01.023) 25762335 PMC4375624

[18] Ouldridge TE. 2015 DNA nanotechnology: understanding and optimisation through simulation. Mol. Phys. **113**, 1-15. (10.1080/00268976.2014.975293)

[19] Holliday R. 1964 A mechanism for gene conversion in fungi. Genet. Res. **5**, 282-304. (10.1017/S0016672300001233) 18976517

[20] Jinek M, Chylinski K, Fonfara I, Hauer M, Doudna JA, Charpentier E. 2012 A programmable dual-RNA-guided DNA endonuclease in adaptive bacterial immunity. Science **337**, 816-821. (10.1126/science.1225829) 22745249 PMC6286148

[21] Hong F, Šulc P. 2019 Strand displacement: a fundamental mechanism in RNA biology? (http://arxiv.org/abs/1811.02766)

[22] Kar S, Baumann WT, Paul MR, Tyson JJ. 2009 Exploring the roles of noise in the eukaryotic cell cycle. Proc. Natl Acad. Sci. USA **106**, 6471-6476. (10.1073/pnas.0810034106) 19246388 PMC2672517

[23] Vilar JMG, Kueh HY, Barkai N, Leibler S. 2002 Mechanisms of noise-resistance in genetic oscillators. Proc. Natl Acad. Sci. USA **99**, 5988-5992. (10.1073/pnas.092133899) 11972055 PMC122889

[24] Plesa T, Zygalakis KC, Anderson DF, Erban R. 2018 Noise control for molecular computing. J. R. Soc. Interface **15**, 20180199. (10.1098/rsif.2018.0199) 29997258 PMC6073653

[25] Plesa T, Erban R, Othmer HG. 2018 Noise-induced mixing and multimodality in reaction networks. Eur. J. Appl. Math. **30**, 887-911. (10.1017/S0956792518000517)

[26] Erban R, Chapman J 2019 Stochastic modelling of reaction–diffusion processes. Cambridge Texts in Applied Mathematics. Cambridge, UK: Cambridge University Press.

[27] Plesa T, Vejchodský T, Erban R. 2016 Chemical reaction systems with a homoclinic bifurcation: an inverse problem. J. Math. Chem. **54**, 1884-1915. (10.1007/s10910-016-0656-1)

[28] Plesa T. 2021 Stochastic approximation of high- by bi-molecular reactions. (http://arxiv.org/abs/1811.02766)

[29] Drengstig T, Ueda HR, Ruoff P. 2008 Predicting perfect adaptation motifs in reaction kinetic networks. J. Phys. Chem. B **112**, 16 752-16 758. (10.1021/jp806818c) 19367864

[30] Ferrell JE. 2016 Perfect and near-perfect adaptation in cell signaling. Cell Syst. **2**, 62-67. (10.1016/j.cels.2016.02.006) 27135159

[31] Chandra FA, Buzi G, Doyle JC. 2011 Glycolytic oscillations and limits on robust efficiency. Science **333**, 187-192. (10.1126/science.1200705) 21737735

[32] Barkai N, Leibler S. 1997 Robustness in simple biochemical networks. Nature **387**, 913-917. (10.1038/43199) 9202124

[33] Spiro P, Parkinson J, Othmer HG. 1997 A model of excitation and adaptation in bacterial chemotaxis. Proc. Natl Acad. Sci. USA **94**, 7263-7268. (10.1073/pnas.94.14.7263) 9207079 PMC23809

[34] Yi TM, Huang Y, Simon MI, Doyle J. 2000 Robust perfect adaptation in bacterial chemotaxis through integral feedback control. Proc. Natl Acad. Sci. USA **97**, 4649-4653. (10.1073/pnas.97.9.4649) 10781070 PMC18287

[35] Gardner TS, Cantor CR, Collins JJ. 2000 Construction of a genetic toggle switch in *Escherichia coli*. Nature **403**, 339–342. (10.1038/35002131) 10659857

[36] Qu Z, Garfinkel A, Weiss JN, Nivala M. 2011 Multi-scale modeling in biology: how to bridge the gaps between scales? Prog. Biophys. Mol. Biol. **107**, 21-31. (10.1016/j.pbiomolbio.2011.06.004) 21704063 PMC3190585

[37] Kepler TB, Elston TC. 2001 Stochasticity in transcriptional regulation: origins, consequences, and mathematical representations. Biophys. J. **81**, 3116-3136. (10.1016/S0006-3495(01)75949-8) 11720979 PMC1301773

[38] Plesa T, Vejchodský T, Erban R. 2017 Test models for statistical inference: two-dimensional reaction systems displaying limit cycle bifurcations and bistability. In *Stochastic processes, multiscale modeling, and numerical methods for computational cellular biology* (ed. D Holcman), pp. 3–27. Cham, Switzerland: Springer International Publishing.

[39] Briat C, Gupta A, Khammash M. 2016 Antithetic integral feedback ensures robust perfect adaptation in noisy bimolecular networks. Cell Syst. **2**, 15-26. (10.1016/j.cels.2016.01.004) 27136686

[40] Qian Y, Del Vecchio D. 2018 Realizing ‘integral control’ in living cells: how to overcome leaky integration due to dilution? J. R. Soc. Interface **15**, 20170902. (10.1098/rsif.2017.0902) 29436515 PMC5832733

[41] Del Vecchio D, Abdallah H, Qian Y, Collins JJ. 2017 A blueprint for a synthetic genetic feedback controller to reprogram cell fate. Cell Syst. **4**, 109-120. (10.1016/j.cels.2016.12.001) 28065574 PMC5326680

[42] Bruno S, Al-Radhawi MA, Sontag ED, Del Vecchio D. 2019 Stochastic analysis of genetic feedback controllers to reprogram a pluripotency gene regulatory network. In *2019 American Control Conference (ACC), 10–12 July, Philadelphia, PA*, pp. 5089–5096. New York, NY: IEEE. (10.23919/ACC.2019.8814355)

[43] Briat C, Gupta A, Khammash M. 2016 Antithetic proportional-integral feedback for reduced variance and improved control performance of stochastic reaction networks. J. R. Soc. Interface **15**, 20180079. (10.1098/rsif.2018.0079) PMC6030643 29899158

[44] Laurenti L, Kwiatkowska M, Czikasz-Nagy A, Cardelli L. 2018 Molecular filters for noise reduction. Biophys. J. **114**, 3000-3011. (10.1016/j.bpj.2018.05.009) 29925035 PMC6026371

[45] Kurtz TG. 1972 The relationship between stochastic and deterministic models for chemical reactions. J. Chem. Phys. **57**, 2976-2978. (10.1063/1.1678692)

[46] Norman TM, Lord ND, Paulsson J, Losick R. 2015 Stochastic switching of cell fate in microbes. Annu. Rev. Microbiol. **69**, 381-403. (10.1146/annurev-micro-091213-112852) 26332088

[47] Bressloff PC. 2017 Stochastic switching in biology: from genotype to phenotype. J. Phys. A: Math. Theor. **50**, 133001. (10.1088/1751-8121/aa5db4)

[48] Balaban NQ, Merrin J, Chait R, Kowalik L, Leibler S. 2004 Bacterial persistence as a phenotypic switch. Science **305**, 1622-1625. (10.1126/science.1099390) 15308767

[49] Feinberg M. 1979 *Lectures on chemical reaction networks*, Delivered at the Mathematics Research Center, University of Wisconsin. See http://www.chbmeng.ohio-state.edu/∼FEINBERG/LecturesOnReactionNetworks/.

[50] Gillespie DT. 1977 Exact stochastic simulation of coupled chemical reactions. J. Phys. Chem. **81**, 2340-2361. (10.1021/j100540a008)

[51] King GAM. 1983 Reactions for chemical systems far from equilibrium. J. Chem. Soc., Faraday Trans. 1 **79**, 75-80. (10.1039/f19837900075)

[52] Deshpande A, Ouldridge TE. 2019 High rates of fuel consumption are not required by insulating motifs to suppress retroactivity in biochemical circuits. Eng. Biol. **1**, 86-99. (10.1049/enb.2017.0017)

[53] Deshpande A, Ouldridge TE. 2019 Optimizing enzymatic catalysts for rapid turnover of substrates with low enzyme sequestration. In the submission process. (http://arxiv.org/abs/1905.00555)

[54] Machinek RR, Ouldridge TE, Haley NE, Bath J, Turberfield AJ. 2014 Programmable energy landscapes for kinetic control of DNA strand displacement. Nat. Commun. **5**, 5324. (10.1038/ncomms6324) 25382214

[55] Cisse II, Kim H, Ha T. 2012 A rule of seven in Watson–Crick base-pairing of mismatched sequences. Nat. Struct. Mol. Biol. **19**, 623-627. (10.1038/nsmb.2294) 22580558 PMC3372693

[56] McKinney SA, Déclais A -C, Lilley DMJ, Ha T. 2003 Structural dynamics of individual Holliday junctions. Nat. Struct. Biol. **10**, 93-97. (10.1038/nsb883) 12496933

[57] Kempter S, Khmelinskaia A, Strauss MT, Schwille P, Jungmann R, Liedl T, Bae W. 2019 Single particle tracking and super-resolution imaging of membrane-assisted stop-and-go diffusion and lattice assembly of DNA origami. ACS Nano **13**, 996-1002. (10.1021/acsnano.8b04631) 30588792

[58] Chen X. 2012 Expanding the rule set of DNA circuitry with associative toehold activation. J. Am. Chem. Soc. **134**, 263-271. (10.1021/ja206690a) 22129141 PMC3260326

[59] Cisse I, Okumus B, Joo C, Ha T. 2007 Fueling protein–DNA interactions inside porous nanocontainers. Proc. Natl Acad. Sci. USA **104**, 12 646-12 650. (10.1073/pnas.0610673104) PMC1937520 17563361

[60] Scalise D, Dutta N, Schulman R. 2018 DNA strand buffers. J. Am. Chem. Soc. **140**, 12 069-12 076. (10.1021/jacs.8b05373) 30204433

[61] Pavliotis GA, Stuart AM. 2008 Multiscale methods: averaging and homogenization. New York, NY: Springer.

[62] Young KD, Kokotovic P, Utkin V. 1977 A singular perturbation analysis of high-gain feedback systems. EEE Trans. Automat. Control **22**, 931-938. (10.1109/TAC.1977.1101661)

[63] Cappelletti D, Ortiz-Munoz A, Anderson DF, Winfree E. 2020 Stochastic chemical reaction networks for robustly approximating arbitrary probability distributions. Theoretical Comput. Sci. **801**, 64-95. (10.1016/j.tcs.2019.08.013)

[64] Artyomov MN, Mathur M, Samoilov MS, Chakraborty AK. 2009 Stochastic bimodalities in deterministically monostable reversible chemical networks due to network topology reduction. J. Chem. Phys. **131**, 195103. (10.1063/1.3264948) 19929080 PMC2792330

[65] Del Vecchio D, Ninfa AJ, Sontag ED. 2008 Modular cell biology: retroactivity and insulation. Mol. Syst. Biol. **4**, 161. (10.1038/msb4100204) 18277378 PMC2267736

[66] Kocabey S, Kempter S, List J, Xing Y, Bae W, Schiffels D, Shih WM, Simmel FC, Liedl T. 2015 Membrane-assisted growth of DNA origami nanostructure arrays. ACS Nano **9**, 3530-3539. (10.1021/acsnano.5b00161) 25734977 PMC4415451

[67] Képès F. 2004 Periodic transcriptional organization of the *E. coli* genome. J. Mol. Biol. **340**, 957-964. (10.1016/j.jmb.2004.05.039) 15236959

[68] Kolesov G, Wunderlich Z, Laikova ON, Gelfand MS, Mirny LA. 2007 How gene order is influenced by the biophysics of transcription regulation. Proc. Natl Acad. Sci. USA **104**, 13 948-13 953. (10.1073/pnas.0700672104) PMC1955771 17709750

[69] Kuhlman TE, Cox EC. 2012 Gene location and DNA density determine transcription factor distributions in *Escherichia coli*. Mol. Syst. Biol. **8**, 610. (10.1038/msb.2012.42) 22968444 PMC3472691

[70] Pulkkinen O, Metzler R. 2013 Distance matters: the impact of gene proximity in bacterial gene regulation. Phys. Rev. Lett. **110**, 198101. (10.1103/PhysRevLett.110.198101) 23705743

[71] Grebenkov DS, Metzler R, Oshanin G. 2018 Strong defocusing of molecular reaction times results from an interplay of geometry and reaction control. Commun. Chem. **1**, 96. (10.1038/s42004-018-0096-x)

[72] Atitey K, Loskot P, Rees P. 2018 Determining the transcription rates yielding steady-state production of mRNA in the lac genetic switch of *Escherichia coli*. J. Comput. Biol. **25**, 1023-1039. (10.1089/cmb.2018.0055) 29957031

[73] Milo R, Phillips R. 2015 Cell biology by the numbers. New York, NY: Garland Science.

[74] Chen YI *et al.* 2019 Measuring DNA hybridization kinetics in live cells using a time-resolved 3D single-molecule tracking method. J. Am. Chem. Soc. **141**, 15 747-15 750. (10.1021/jacs.9b08036) PMC7179573 31509386

[75] Bhadra S, Ellington AD. 2014 Design and application of cotranscriptional non-enzymatic RNA circuits and signal transducers. Nucleic Acids Res. **42**, e58. (10.1093/nar/gku074) 24493736 PMC3985647

